# Motivational Profiles and Associations With Physical Activity Before, During, and After the COVID-19 Pandemic: Retrospective Study

**DOI:** 10.2196/43411

**Published:** 2023-04-24

**Authors:** Kayla Nuss, Wuyou Sui, Ryan Rhodes, Sam Liu

**Affiliations:** 1 Klein Buendel Golden, CO United States; 2 School of Exercise Science, Physical & Health Education University of Victoria Victoria, BC Canada

**Keywords:** motivational profile, self-determination theory, physical activity, COVID-19, health lifestyle, latent profile analysis, mental well-being, health intervention

## Abstract

**Background:**

In March 2020, the World Health Organization declared the worldwide COVID-19 outbreak a pandemic, triggering many countries, including Canada, to issue stay-at-home orders to their citizens. Research indicates that these stay-at-home orders are associated with a decline in physical activity (PA), a behavior that can reduce disease risk and improve the quality of life. Many behavioral change theories, such as the self-determination theory (SDT) of motivation, state that PA engagement is mediated by psychological constructs, such as motivation. According to the SDT, motivation exists on a continuum from more controlled (external or coerced) to more autonomous (volitional) regulatory forms. Individuals move along the continuum from more controlled to more autonomous forms through the fulfillment of 3 psychological needs: autonomy, competence, and relatedness. Research indicates that moderate-to-vigorous physical activity (MVPA) is positively associated with the autonomous regulatory form of motivation. Recently, researchers have speculated that a better method to describe motivation than movement along the continuum is to generate motivational profiles, which represent combinations of differing levels of controlled and autonomous regulation existing simultaneously.

**Objective:**

We aimed to identify distinct motivational profiles and determine their association with MVPA before, during, and after the COVID-19 pandemic.

**Methods:**

Using a cross-sectional, retrospective design, we surveyed 977 Canadian adults. We assessed motivation for PA using the Behavioral Regulations in Exercise Questionnaire-3 (BREQ-3). We assessed PA pre–, during, and post–COVID-19 stay-at-home orders in Canada using the International Physical Activity Questionnaire (IPAQ). We derived motivational profiles using latent profile analysis (LPA). Using motivational profiles as an independent variable, we assessed their effect on PA at all 3 time points with multilevel models that included the participant ID as a random variable.

**Results:**

We identified 4 profiles: high controlled and high autonomous (HCHA), low overall motivation (LOM), high autonomous and introjected (HAI), and high amotivation and external (HAE). The HCHA profile had the highest levels of weekly MVPA minutes at all 3 time points, followed by the HAI profile.

**Conclusions:**

Our results suggest that a combination of both autonomous and controlled regulatory forms may be more effective in influencing MVPA than the controlled or autonomous forms alone, particularly during times of high stress, such as a worldwide pandemic. Although the odds of another global pandemic are low, these results may also be applied to other times of stress, such as job transitions, relationship changes (eg, change in marital status), or the death of a loved one. We suggest that clinicians and practitioners consider developing PA interventions that seek to increase both controlled and autonomous regulatory forms instead of aiming to reduce controlled forms.

## Introduction

In late 2019, several community members from Wuhan, Hubei Province, in China fell ill from a novel coronavirus [1]. On January 30, 2020, the World Health Organization declared the outbreak to be a public health emergency and on March 11, 2020, a pandemic [1]. As a result, several countries, including Canada, instructed citizens to stay at home and maintain physical distance from each other.

Early evidence suggests that the pandemic and associated stay-at-home orders had a detrimental effect on health behaviors, such as physical activity (PA) [2,3]. The Canadian 24-Hour Movement Guidelines for Adults recommend at least 150 minutes of cumulative moderate-to-vigorous physical activity (MVPA) per week, as much light-intensity PA as possible, and frequent interruption of sedentary time [4]. Abiding by these guidelines is associated with lower overall disease risk and improved quality of life and overall health [4]. Despite these benefits, however, several studies indicate that Canadians participated in fewer minutes of MVPA during the first stay-at-home orders issued during the COVID-19 pandemic [5-7].

Due to the incongruence between the benefits of MVPA and MVPA participation, it is imperative that clinicians and practitioners identify strategies to increase MVPA overall, especially during stressful periods, such as a worldwide health crisis. Such strategies might include behavioral change techniques, such as external pressure, coercion, rewards, or incentives [8]. Lasting behavioral change, however, is more likely to occur if the underlying psychological constructs are affected. For example, self-determination theory (SDT), a frequently used framework to understand PA motivation, asserts that motivation is grounded in the fulfilment of 3 psychological needs: autonomy, competence, and relatedness [9]. When the psychological needs are satisfied, people can move along the motivation continuum, from more controlled forms of regulatory subtypes (external or coerced) to more autonomous (volitional) forms of regulatory subtypes.

At 1 end of the motivation continuum lies amotivation, which describes a state in which the individual is nonautonomous and has no drive for the behavior [10]. Following amotivation are the extrinsic forms, the most extreme of which is external regulation. Externally regulated behaviors stem from a desire to be compliant, to conform, or to receive external rewards or avoid external punishments [10]. For example, a person who wants praise or recognition from their physician may be more externally regulated for PA. Moving along the continuum, introjected regulation refers to motivation that is grounded in a drive for self-control, a need to protect one’s ego, or receive internal rewards (eg, feeling pride), or avoid internal punishments (eg, feeling guilt) [10]. Identified regulation of behavior occurs when motivation is somewhat internal and is based on conscious values. Someone who values their health and recognizes that PA can contribute to health may have higher identified regulation for PA. The most internalized form of extrinsic motivation is integrated regulation, which is driven by intrinsic sources, such as the desire to act in congruence with one’s values and sense of self. For example, a person with high integrated regulation for PA may consider PA as a component of their identity; they are a person who performs PA regularly. Intrinsic motivation is the most autonomous form, with behaviors grounded in interest, enjoyment, and a sense of satisfaction [10]. In other words, PA is fun and enjoyable; therefore, someone with high intrinsic motivation engages in it.

It may not be possible for every person to become intrinsically motivated to perform PA, but it is possible for individuals to move from amotivation to more autonomous engagement in PA [11]. A large body of research has identified that more autonomous forms of motivation (ie, intrinsic and more internalized forms of extrinsic motivation) are associated with engagement in MVPA [9]. Specifically, identified motivation predicts PA adoption, and intrinsic motivation predicts long-term participation [12]. Additionally, a robust meta-analysis of the effect of SDT grounded interventions on health behaviors found that autonomous regulatory forms (ie, identified and integrated regulation, and intrinsic motivation) are associated with increases in both self-reported and objectively assessed MVPA [13]. Furthermore, the same meta-analysis identified a strong association between the psychological need of perceived competence and autonomous regulatory forms, adding support to the overall SDT model of motivation for health behaviors [13].

Traditionally, motivation for PA has been quantified using the 6 different regulatory subtype scores (ie, amotivation, external regulation, introjected regulation, identified regulation, integrated regulation, and intrinsic motivation). In research, these individual variable scores are then compared to outcome variables, such as PA, to conclude that people with high scores on, for example, integrated regulation are also more likely to participate in PA [14]. This approach ignores the possibility that some individuals with high integrated regulation may also have high scores on introjected or external regulation or low scores on intrinsic motivation. As demonstrated by Chemolli and Gagné [15], individuals can report high levels of both more controlled and more autonomous forms of regulatory subtypes. Perhaps then, a better way to conceptualize motivation is as a multidimensional construct that varies in regulatory subtype magnitude rather than an overall degree of relative autonomy [14]. Identifying motivational profiles in a population is emerging as a more appropriate means of classifying motivation for PA, and latent profile analysis (LPA) is 1 of several methods to identify distinct motivational profiles among a sample [14,16].

In the context of a global pandemic, it is particularly important to investigate motivation for PA as a multidimensional construct because of the unprecedented conditions that the associated stay-at-home orders created. For example, if an individual had a high level of external regulation for PA due to external pressure from family or friends, when attention turned to working from home, educating children at home, and avoiding contracting the virus, did that external pressure persist? In contrast, that individual may also have had a substantial dose of integrated regulation, whereby they also identify as a person who regularly engages in PA. In this case, this person may persist with PA engagement, even in the absence of external pressure. Without examining the interplay of within-person motivational types, it is challenging to discern who may persist with PA engagement, particularly in times of uncertainty, such as a worldwide pandemic. Since PA levels decreased in Canada during the pandemic [5-7], we aimed to determine whether specific motivational profiles were protective against these decreases and whether certain profiles predicted a more rapid return to MVPA. We anticipate that these results may benefit clinicians and practitioners to target particular groups (eg, groups for which motivational profiles predict a decline in MVPA) during stressful periods in order to maintain MVPA levels.

Therefore, the first objective of this study was to use LPA to identify the motivational profiles for PA among a sample of Canadian adults. Based on previous research, we hypothesized that we would identify common profiles marked by (1) high amotivation and external regulation, (2) high autonomous forms (ie, identified and integrated regulation, intrinsic motivation), and (3) low on all regulatory forms, as well as some profiles specific to our sample [14,17].

The second objective was to evaluate the association between motivational profiles for PA and the average weekly minutes of MVPA pre–COVID-19 (prior to March 2020), during the most recent COVID-19–associated stay-at-home orders (early January-early March 2022), and after much of Canada lifted those orders (March 2022). We hypothesized that those individuals with profiles characterized by greater autonomous motivation would engage in higher mean levels of daily MVPA compared to those with more external or controlled motivational profiles for PA at all 3 time points. Controlled forms of motivation can be easily influenced by one’s environment [18]. Therefore, those with more externally controlled PA behaviors were hypothesized to reduce their engagement during the COVID-19 stay-at-home orders, due to the closing of fitness facilities and increase them once the lockdowns were lifted.

## Methods

### Participants and Procedure

We conducted a retrospective survey that took place between March 11 and 16, 2022. Of the 1205 respondents, 977 (81.1%) provided informed consent, completed the survey, and were adults between the ages of 18 and 74 years, living in Canada. The minimum completion time was 5 minutes, and the median completion time was 21 minutes. A third-party market research company Maru/Blue was used to recruit participants. Maru/Blue has an online polling database of 120,000 Canadians with a representative sample compared to the Canadian census [19,20]. Panel participants are recruited through a variety of online and offline methods and receive small cash incentives and prize opportunities from the company after completing surveys.

### Ethical Considerations

Prior to recruiting participants, ethics approval was obtained from the Institutional Ethics Board of University of Victoria (#21-0405). Participants were presented with an informed consent statement on the front page of the electronic survey. Only those who consented were allowed to proceed to the survey items. Informed consent was obtained from all participants. Participants were not compensated for survey participation or completion by our research team but may have been compensated by Maru/Blue for survey completion. These data were not shared with the research team. Survey responses were anonymous as no potentially identifying data were collected in this study.

### Measures

Demographics, including age, sex, education, ethnicity, employment status, and income, were collected using self-report measures. Motivation for PA was assessed using the Behavioral Regulation in Exercise Questionnaire-3 (BREQ-3), which has been validated for use in this population [21,22]. The BREQ-3 assesses motivation for exercise using the 6 SDT regulatory subtypes: amotivation, external regulation, introjected regulation, identified regulation, integrated regulation, and intrinsic motivation. Each subtype is assessed on a 5-point Likert scale ranging from 0 to 4, where 0=“not true for me” and 4=“very true for me.” We calculated the mean scores for each regulatory subtype and used those values to identify distinct motivational profiles. In this study, the Cronbach α (95% CI) values for the internal consistency for each motivational subtype were as follows: amotivation: 0.91 (0.90-0.93); external regulation: 0.88 (0.87-0.90); introjected regulation: 0.88 (0.86-0.89); identified regulation: 0.83 (0.81-0.85); integrated regulation: 0.89 (0.87-0.91); and intrinsic motivation: 0.93 (0.92-0.94).

To assess PA at 3 time points (pre–COVID-19, prior to March 2020; during the most recent COVID-19 stay-at-home orders in Canada, January-early March 2022; and in the past 7 days, March 2022), participants responded to items from the International Physical Activity Questionnaire (IPAQ) [23]. Participants were asked to report the number of hours per week that they participated in vigorous, moderate, and mild PA. We summed the hours of vigorous and moderate PA and multiplied the total by 60 minutes to obtain a value for MVPA minutes at all 3 time points.

### Statistical Analyses

To account for extreme outliers, we chose to perform 95% Winsorization on the minutes of MVPA per week at all 3 time points. We also used the Mahalanobis distance with a *P* value of .001 as a cutoff to identify multivariate outliers for the motivational subtype variables. After identifying these outliers, we performed LPA, both with and without them in the sample. We chose to proceed with the multivariate outliers included in the sample as the outcomes were the same with and without them. We then calculated means and SDs for continuous demographic variables and frequencies and percentages for categorical variables.

### Latent Profile Analysis

To derive motivational profiles, we used LPA. To obtain the most appropriate model and number of classes, LPA uses a number of quantitative fit statistics, including the log-likelihood, the Akaike information criterion (AIC), the Bayesian information criterion (BIC), and entropy [14]. Higher log-likelihood values and lower AIC and BIC values indicate a better model fit [24]. Entropy represents the probability that members of a sample are able to fit into 1 of the resulting classes or profiles, and the magnitude of difference between classes. An entropy value of 1 would indicate that 100% of the sample fits into 1 of the classes; therefore, entropy values close to 1 are preferred [25]. After considering fit statistics, we examined each model to determine which one contained a set of substantive and distinct profiles with differing levels of each subtype.

We used participant mean scores for each motivation subscale to calculate *z*-scores, which were then used to conduct LPA. After choosing the model with the best quantitative and qualitative fit both, we conducted 1-way ANOVA to determine differences in each motivational subtype among the resultant classes. When the overall main effects of class were detected, we used the Tukey honestly significant difference (HSD) test for pairwise comparisons between classes. We also calculated the partial η^2^ to determine the effect sizes of the profile on each motivational subtype.

### Multilevel Regression Models

To examine the effects of motivational profiles on the weekly minutes of PA pre–COVID-19, during the COVID stay-at-home orders, and in the past 7 days, we used multilevel regression models using the participant ID as a random variable. We added biological sex, age, ethnicity, education, income, and current employment as covariates to the model. We examined the interaction between class and time (pre–COVID-19, during COVID-19, and in the past 7 days). When the overall main effects of class were detected, we used the Tukey HSD test for pairwise comparisons between classes. We also calculated partial η^2^ to determine effect sizes of the class on MVPA. We used the benchmarks defined by Cohen for small (η^2^=0.01), medium (η^2^=0.06), and large (η^2^=0.14) effect sizes for interpretation. We used R version 4.1.3 (R Foundation for Statistical Computing), and statistical significance was set to *P*<.01 for all analyses.

## Results

### Participant Details

The final sample consisted of 977 adults living in Canada in March 2022. The mean age of sample participants was 46.79 (SD 15.23) years. The majority of the sample identified as female (n=497, 50.9%), were of European (n=408, 41.8%) or other North American (n=374, 38.3%) origins, were employed full-time (n=537, 55.0%), had at least a bachelor’s degree (n=488, 49.9%), and had an income above CA $80,000 (US $58,358) per year (n=564, 57.7%).

### Motivational Profiles

Using quantitative fit statistics log-likelihood, AIC, BIC, and entropy, we determined that the model of best fit was either a 4-, 5-, or 6-class option. Although the 5- and 6-class solutions had lower AIC and BIC values, we determined that the 4-class solution contained qualitatively distinct profiles that better represent the full spectrum of motivational subtypes of SDT. Both 5- and 6-class solutions contained profiles that were similar to other resulting profiles in the same model (see Table 1).

**Figure 1:**
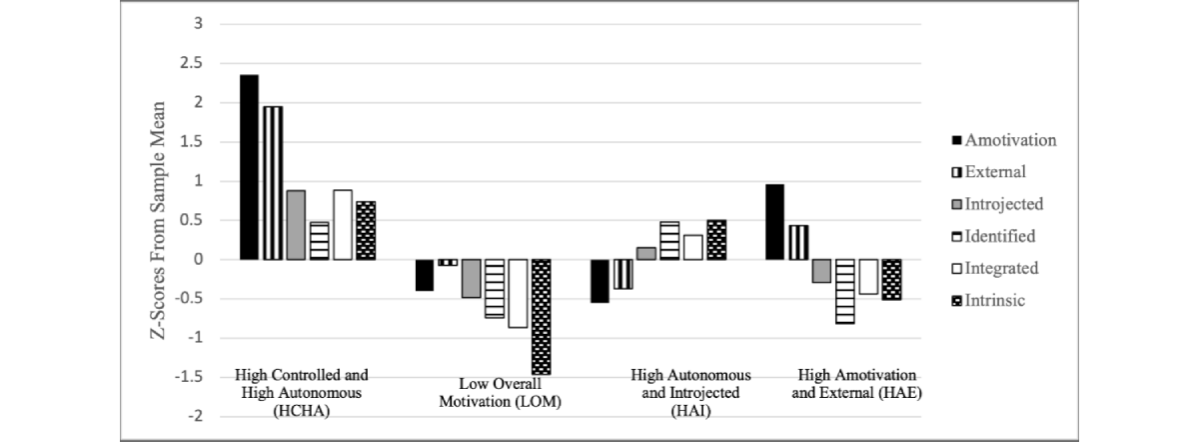
Motivational profiles: z-scores depicting distinct differences in motivational subtypes from sample means. Mot.: motivation; Reg.: regulation.

Figure 1 shows the 4 resulting profiles as depicted by their motivational subtype z-scores. These scores indicate how much each profile differs from the individual subtype sample means. In this figure, 0 on the y axis represents the sample mean for each distinct motivational subtype. The “high controlled and high autonomous” (HCHA) regulation profile (n=56, 5.7%) had higher-than-expected scores on all 6 motivational subtypes but relatively higher controlled versus autonomous scores compared to the sample mean. The “low overall motivation” (LOM) profile (n=130, 13.3%) had lower-than-expected scores on all motivational subtypes. The “high autonomous and introjected” (HAI) regulation profile (n=566, 57.9%) had higher-than-expected scores on introjected regulation and the autonomous motivational subtypes. The HAE regulation profile (n=225, 23.0%) had higher-than-expected scores on both amotivation and external regulation and lower-than-expected scores on autonomous subtypes.

Table 2 shows the differences in motivational subtypes among each of the 4 profiles. We detected the main effect of profile on amotivation (*F*_3,973_=1067.7, *P*<.01, 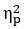 =0.77), external regulation (*F*_3,973_=171.40, *P*<.01, 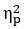
=0.35), introjected regulation (*F*_3,973_=48.16, *P<*.01, 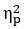
=0.13), identified regulation (*F*_3,973_=234.96, *P<*.01, 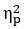
=0.42), integrated regulation (*F*_3,973_=120.57, *P<*.01, 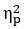
=0.27), and intrinsic regulation (*F*_3,973_=437.07, *P<.*01, 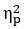
=0.57). Pairwise comparisons revealed that HCHA is significantly different than LOM and HAE or all subtypes and significantly different than HAI for all subtypes except identified regulation. LOM is significantly different than HAI for all subtypes and is significantly different than HAE for all subtypes except introjected regulation and identified regulation. HAI and HAE are significantly different for all regulatory subtypes.

**Table 1:** Motivational profile model fit statistics for latent profile models.

Number of classes	Log-likelihood	AIC^a^	BIC^b^	Entropy
4^c^	–6511.70	13,119.40	13,353.86	0.84
5	–6451.75	13,013.50	13,282.15	0.82
6	–6349.14	12,822.27	13,125.11	0.82

^a^AIC: Akaike information criterion.

^b^BIC: Bayesian information criterion.

^c^The 4-class model was selected based on both quantitative and qualitative fit statistics.

**Table 2:** Differences in motivational subtypes among the 4 motivational profiles.

Motivational subtype	Overall, mean (SD)	HCHA^a^, mean (SD)	LOM^b^, mean (SD)	HAI^c^, mean (SD)	HAE^d^, mean (SD)
Amotivation	0.92 (0.92)	3.10 (0.48)	0.55 (0.48)^e^	0.42 (0.44)^e,f^	1.87 (0.43)^e,f,g^
External regulation	1.21 (0.97)	3.13 (0.50)	1.13 (0.98)^e^	0.86 (0.75)^e,f^	1.65 (0.82)^e,f,g^
Introjected regulation	2.13 (1.02)	3.06 (0.50)	1.54 (1.19)^e^	2.30 (0.98)^e,f^	1.82 (0.79)^e,g^
Identified regulation	2.74 (0.78)	3.11 (0.48)	2.07 (0.84)^e^	3.12 (0.54)^f^	2.06 (0.59)^e,g^
Integrated regulation	2.16 (1.00)	3.05 (0.59)	1.22 (0.84)^e^	2.47 (0.90)^e,f^	1.71 (0.79)^e,f,g^
Intrinsic motivation	2.36 (0.98)	3.11 (0.48)	0.82 (0.58)^e^	2.85 (0.64)^e,f^	1.82 (0.71)^e,f,g^

^a^HCHA: high controlled and high autonomous.

^b^LOM: low overall motivation.

^c^HAI: high autonomous and introjected.

^d^HAE: high amotivation and external.

^e^Significantly different from HCAC.

^f^Significantly different from LOM.

^g^Significantly different from HAI.

### Effects of Motivational Profiles on Weekly Minutes of MVPA

**Figure 2:**
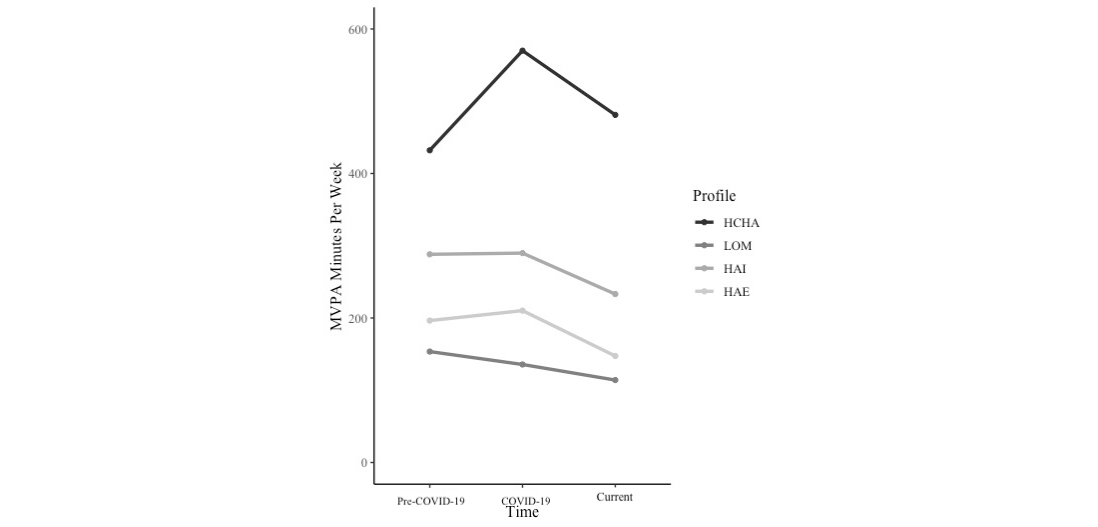
Minutes of MVPA per week by motivational profile pre–COVID-19, during COVID-19, and within the past 7 days. HAE: high amotivation and external; HAI: high autonomous and introjected; HCHA: high controlled and high autonomous; LOM: low overall motivation; MVPA: moderate-to-vigorous physical activity.

Table 3 shows the means and SDs for weekly minutes of MVPA by profile pre–COVID-19, during COVID-19, and in the past 7 days. Figure 2 shows the results of the multilevel model with minutes of MVPA pre–COVID-19, during COVID-19, and in the past 7 days as the outcome variable. We detected a significant interaction between profile and time (*F*_6,1945.03_=3.08, *P*=.005, 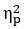
=0.01). According to Agerström et al [26], the resulting η^2^ represents a small, but meaningful, effect size. Pairwise comparisons revealed that LOM, HAI, and HAE had significantly fewer hours of MVPA at all 3 time points compared to HCHA. LOM and HAE had significantly fewer minutes of MVPA at all 3 time points compared to HAI. No significant differences in the minutes of MVPA were detected between LOM and HAE at any time point.

**Table 3:** Weekly minutes of MVPA^a^ pre–COVID-19, during COVID-19, and in the past 7 days.

Time point	Overall, mean (SD)	HCHA^b^, mean (SD)	LOM^c^, mean (SD)	HAI^d^, mean (SD)	HAE^e^, mean (SD)
Pre–COVID-19	257.50 (326.33)	448.39 (365.46)	146.31 (303.41)^f^	289.19 (324.76)^f,g^	194.67 (298.93)^f,h^
During COVID-19	267.08 (350.30)	586.34 (437.23)	128.42 (276.52)^f^	290.64 (345.21)^f,g^	208.47 (318.75)^f,h^
Past 7 days	211.77 (323.35)	497.41 (423.36)	106.85 (265.41)^f^	233.88 (321.34)^f,g^	145.67 (279.53)^f,h^

^a^MVPA: moderate-to-vigorous physical activity.

^b^HCHA: high controlled and high autonomous.

^c^LOM: low overall motivation.

^d^HAI: high autonomous and introjected.

^e^HAE: high amotivation and external.

^f^Significantly different from HCAC.

^g^Significantly different from LOM.

^h^Significantly different from HAI.

## Discussion

### Principal Findings

The first aim of this study was to identify the motivational profiles for PA in a sample of 977 Canadian adults. We hypothesized that we would detect profiles identified in other PA motivation research characterized by high amotivation and external regulation, high autonomous forms (ie, identified and integrated regulation, and intrinsic motivation), and low on all forms of regulation [14,17]. The study identified 4 distinct motivational profiles for PA in this sample, confirming our first hypothesis. The resulting profiles were high controlled and high autonomous (HCHA), low overall motivation (LOM), high autonomous and introjected (HAI), and high amotivation and external (HAE) regulation. Other studies assessing motivational profiles and PA have identified profiles characterized by high z-scores on autonomous regulatory forms and low z-scores on controlled forms, profiles characterized by high z-scores on controlled regulatory forms and low z-scores on autonomous forms, or combinations of all high or all low z-scores [16,17,27-33]. Although other studies have identified profiles with both HCHA regulatory forms [16,31], to the best of our knowledge, only Gourlan et al [16] also identified a profile with elevated amotivation, controlled regulatory subtypes, and autonomous regulatory subtypes.

The second aim of this study was to investigate the association between resulting motivational profiles and MVPA pre–COVID-19, during the most recent COVID-19–related stay-at-home orders in Canada (early 2022), and after those orders had been lifted (March 2022). Our second hypothesis was that specific profiles would predict MVPA participation at all 3 time points. We also detected a significant effect of profile on MVPA at all 3 time points. Specifically, HCHA, HAI, and HAE reported more weekly minutes of MVPA during the most recent stay-at-home orders in Canada than their pre–COVID-19 levels, whereas LOM decreased their minutes of MVPA. Further, LOM, HAI, and HAE reported fewer weekly minutes of MVPA in the past 7 days compared to their levels during the COVID-19 lockdown and pre–COVID-19. HCHA, however, reported a decrease in MVPA in the past 7 days in comparison to COVID-19 levels but an increase in comparison to pre–COVID-19 levels.

We were surprised to see that it was not HAI, with higher-than-average autonomous regulatory subtype scores, with the most minutes of MVPA but rather HCHA regulatory forms. Our findings are inconsistent with the literature in that, typically, amotivation and controlled regulatory forms are not associated with regular PA engagement [14,28,29,34,35]. Furthermore, controlled regulatory forms are heavily reliant on external factors (eg, family and friends who encourage the behavior) for behavioral engagement. These behaviors tend to extinguish once those factors are removed, resulting in lower levels of behavioral persistence [18]. Although controlled regulatory forms may be sufficient to initiate PA behavior, evidence does not suggest they are sufficient to support long-term engagement [36]. Similarly, a systematic review exploring SDT and PA found negative or null effects of controlled motivation on long-term PA maintenance [35].

Specific to MVPA during the COVID-19 pandemic, our results differed from those of Chirico et al [37], who conducted an assessment of motivation using a metric called the relative autonomy index and its association with the intention for MVPA during the lockdowns in Italy in the initial months of the pandemic. They determined that higher and more positive relative autonomy index scores, which represent higher scores on more autonomous regulatory forms of motivation, predicted the intention to perform PA. In our sample, the HAI profile would likely have the highest relative autonomy index score but only was associated with the second-highest levels of MVPA across time. Interestingly, the profile in our sample with the highest levels of MVPA at all 3 time points, HCHA, would likely result in a relative autonomy index score around 0. Chirico et al [37] assessed the intention for MVPA, whereas we assessed self-reported behavior, which we recognize are not synonymous. Although the association between intention and PA behavior is sizeable, it is not perfect, and several studies have identified a significant gap between intention and behavior in the realm of PA research [38].

These findings, then, suggest that the combination of high levels of all regulatory subtypes is somehow a more influential combination that either controlled or autonomous regulatory subtypes alone. Phillips and Johnson [17] were among the first to assess motivation for PA as a multidimensional construct rather than as a single index score or by individual regulatory subtype score. In their work, they determined that the relationship between controlled and autonomous regulatory subtypes and PA engagement is more complicated than originally thought. Traditionally, researchers have recognized high-quality motivation for PA as having lower levels of controlled regulatory forms and higher levels of autonomous regulatory forms. Phillips and Johnson [17], however, discovered that moderate levels of controlled regulatory forms, combined with any amount of autonomous regulatory forms, predict the highest levels of PA engagement. As such, the traditional stance that controlled regulatory forms of motivation are detrimental to PA may be incorrect, as the data presented here, along with the evidence provided by Phillips and Johnson [17], indicate that controlled regulatory forms in combination with autonomous forms may support PA engagement in some individuals. It is unclear as to why a combination of controlled and autonomous regulatory forms seems to be more supportive of PA among some people. We speculate, however, that the potential negative impacts of controlled motivation for PA that have been identified in the literature, such as decreased enjoyment, persistence, and PA intention [39], were buffered by the elevated autonomous forms. Autonomous regulatory forms, incidentally, are associated with increased enjoyment, persistence, and PA intention [39]. Future research should focus on the interplay between controlled and autonomous forms in PA motivation.

Although it remains unclear as to why individuals within the HCHA and HAE profiles increased their minutes of MVPA from pre–COVID-19 to during COVID-19, we speculate that the overall emphasis on PA during the COVID-19 stay-at-home orders may have been a factor. For example, news outlets, social media, and other media sources emphasized the importance of maintaining PA to reduce the impact of a COVID-19 infection [5] Further, despite the closure of in-person fitness facilities, online and virtual offerings from personal trainers and group fitness instructors increased over the course of the stay-at-home order period [40,41]. Potentially, individuals with elevated doses of controlled regulatory forms were subject to the social pressure (via media and social media outlets) to continue being physically active and took advantage of the novel and widely available fitness and exercise resources offered online. Further investigation is necessary to confirm this interpretation of these study results, however.

We identified that our results are applicable to other contexts beyond the life interruptions of the COVID-19 pandemic. In general, clinicians and practitioners may not want to reduce controlled regulatory forms of PA motivation when desiring to influence PA participation [17]. Perhaps interventions intending to increase *both* more controlled and more autonomous regulatory forms may be more beneficial for some groups. Further, future research can focus on how motivational profiles influence PA engagement during other stressful life episodes, such as job transitions, relationship changes (eg, change in marital status), or the death of a loved one. Research indicates that these life changes can negatively impact PA behavior [42]. Perhaps motivational profiles that include both controlled and autonomous regulatory forms of motivation provide resiliency against reductions in PA.

### Limitations

We recognize that this study is not without limitations. First, we implemented a cross-sectional design, limiting our interpretation of the results to association rather than cause and effect. Second, we asked participations to reflect on their MVPA at 3 different time points, including pre–COVID-19, which was more than 2 years prior to survey administration. Despite the use of the IPAQ, a validated self-report instrument for PA assessment [23], self-report instruments are still vulnerable to overreporting of both the time and the intensity of PA behavior [43]. In our case, the mean reported weekly minutes of MVPA were well above the 150-minute-per-week recommendation for health in Canada [4] at all 3 time points. Finally, we assessed motivation regulatory subtypes at the third time point (March 2022) but then asked participants to reflect on MVPA from prior to March 2020 and then in early 2022. A recent study investigated the stability of motivational profiles and MVPA across a 5-year period and discovered that movement between profiles is not only possible but likely [44]. Therefore, to more clearly investigate motivational profiles and their associations with MVPA at different time points, researchers should undertake a robust longitudinal design like that of Emm-Collison et al [44].

### Conclusion

We derived 4 distinct motivational profiles from our sample. These profiles were characterized by HCHA, LOM, HAI, and HAE regulatory forms. Our findings suggest that motivational profiles predicted PA activity engagement before, during, and after the COVID-19 pandemic and associated stay-at-home orders. Participants characterized by high levels of both controlled and autonomous regulatory forms reported the highest levels of MVPA at all 3 time points, whereas all other groups saw slight increases or decreases from pre–COVID-19 to during COVID-19 but then decreased in the past 7 days to below pre–COVID-19 levels. These results suggest that contrary to previously held beliefs about motivation regulatory subtypes, controlled motivation may not be detrimental to MVPA engagement.

## Abbreviations

AIC: Akaike information criterion
BIC: Bayesian information criterion
BREQ-3: Behavioral Regulation in Exercise Questionnaire-3
HAE: high amotivation and external
HAI: high autonomous and introjected
HCHA: high controlled and high autonomous
HSD: honestly significant difference
IPAQ: International Physical Activity Questionnaire
LOM: low overall motivation
LPA: latent profile analysis
MVPA: moderate-to-vigorous physical activity
PA: physical activity
SDT: self-determination theory

## Data Availability

The data sets generated and analyzed during the study are available from the corresponding author upon reasonable request.
